# Genotype Variations and Association between PAI-1 Promoter Region (4G/5G and -844G/A) and Susceptibility to Acute Myocardial Infarction and Chronic Stable Angina

**DOI:** 10.1155/2021/5551031

**Published:** 2021-06-25

**Authors:** Sunil Kumar, Amit Kumar Verma, Vinay Sagar, Ravi Ranjan, Rahul Sharma, Preeti Tomar, Deepti Bhatt, Yamini Goyal, Mohammed A. Alsahli, Ahmad Almatroudi, Saleh A. Almatroodi, Arshad Husain Rahmani, Faris Alrumaihi, Khursheed Muzammil, Kapil Dev, Rakesh Yadav, Renu Saxena

**Affiliations:** ^1^Department of Microbiology, Government Doon Medical College, Dehradun, India; ^2^Department of Biotechnology, JMI, New Delhi, India; ^3^Department of Internal Medicine, PGIMER, Chandigarh, India; ^4^Department of Hematology, AIIMS, New Delhi, India; ^5^Department of Medical Laboratories, College of Applied Medical Sciences, Qassim University, Buraidah, Saudi Arabia; ^6^Department of Public Health, College of Applied Medical Sciences, Khamis Mushayt, King Khalid University, Abha, Saudi Arabia; ^7^Department of Cardiology, AIIMS, New Delhi, India

## Abstract

The present study aimed at investigating the 4G/5G and -844G/A polymorphisms and plasma concentration of PAI-1 in patients with acute myocardial infarction (AMI) and chronic stable angina (CSA) in Indian population. It included 100 patients with AMI and stable angina and 100 healthy controls. All study subjects were typed for two PAI polymorphisms (4G/5G and -844G/A) through PCR-RFLP and level of PAI through ELISA. The comparison of AMI and CSA independently with control in terms of PAI-1 level was statistically significant but not between AMI and CSA. The frequency of 4G/4G and 4G/5G genotype and 4G allele was significantly higher in AMI cases than in control and was found to increase the risk of AMI. There was a significant relationship between 4G/5G polymorphism and AMI risk under the dominant and codominant genotype. The frequency of 4G/4G genotype and 4G allele was significantly higher in CSA cases than in control group and increases the risk of CSA. There was no significant association between 4G/5G polymorphism and CSA risk under recessive, dominant, and codominant models. The genotype and allelic frequencies difference between the cases (AMI and CSA) and control with regard to -844G/A polymorphisms were statistically nonsignificant. Also, we did not detect any significant association of -844G/A polymorphism with AMI and CSA in recessive, dominant, and codominant models. Along with the traditional risk factors, the 4G/5G allele polymorphism is an independent risk factor for the development of AMI. The detection of 4G/5G allele may therefore be helpful in primary prevention. Patients who carry the 4G/5G allele polymorphism have high concentrations of PAI-1, which might be involved in incidents leading to AMI. The present study for the first time revealed significant association of 4G/5G allele polymorphism with high risk of AMI in Indian population and will be helpful in identifying the genetic risk factors associated with AMI and CSA and for better management of diagnostic measures.

## 1. Introduction

Atherothrombotic changes are one of the major causes of cardiovascular morbidity and mortality. Coronary artery disease (CAD) that encompasses chronic stable angina (CSA), unstable angina (USA), and acute myocardial infarction (AMI) has numerous known genetic and environmental risk factors. Risk factors that are well established include smoking, heavy alcohol consumption, hypertension, diabetes, family history, and dyslipidemia and their association with CAD is believed to be largely under genetic control [1, 2].

Plasminogen activator inhibitor-1 (PAI-1) is one of vital regulatory elements of fibrinolytic pathway. It inhibits both tissue and urokinase specific plasminogen inhibitor; thus its raised level seems to promote prothrombotic state and related vascular changes [3]. Several PAI-1 gene polymorphisms have been described in literature; however, only few of them alter the inhibitor level in plasma. A single guanosine deletion/insertion polymorphism (4G or 5G) located in the promoter region of the PAI-1 gene at 675 base pair upstream from the transcription-starting site has a functional role in PAI-1 synthesis and expression [4]. The PAI-1 level is noted to be higher in 4G allele carrier as compared to 5G carrier, as both polymorphic alleles bind to transcription activator, whereas 5G also binds a repressor protein to an overlapping binding site [5]. PAI-1 4G/5G gene polymorphism is believed to unmask thrombotic phenotype in patients with underlying prothrombotic disorders in both arterial and venous beds such as protein S deficiency, factor V Leiden defect, and antiphospholipid antibody syndrome [6–8]. Numerous studies have already been conducted to evaluate the possible sequel of PAI-1 4G/5G gene polymorphism; nevertheless, they reported only conflicting results.

Similarly, there is another polymorphism with nucleotide substitution of guanine for adenine at 844 position results in altered gene expression and plasma PAI-1 levels, possibly by affecting the binding of nuclear proteins to the PAI-1 promoter. Studies assessing -844G/A polymorphisms and their role in prothrombotic states identified outcomes identical to 4G/5G gene polymorphism.

The present study aimed to investigate the 4G/5G and -844G/A polymorphisms and plasma concentration of PAI-1 in patients with CAD, particularly in AMI and CSA, and its comparison with normal control subjects.

## 2. Material and Methods

### 2.1. Study Subjects

This study was conducted in the Department of Hematology, All India Institute of Medical Sciences, New Delhi, India, after obtaining the ethical clearance from the institutional board (Ref.No.IESC/T-252/03.06.2011) and written informed consent was collected from all patients and controls. We enrolled one hundred nonrelated consecutive AMI patients (male : female = 82 : 18; age range = 18 to 75 years) and one hundred nonrelated consecutive SA patients (M : F = 82 : 18; age range = 18 to 75 years) based on American College Cardiology/American Heart Association (ACC/AHA) criteria. Patients exclusively of North-Indian origin were included in the study. Patients on oral anticoagulant or on any medication (antibiotics, aspirin, contraceptives, or steroids) were included in the study only if they were off the medication for a minimum period of two weeks (from the time of sampling). Patients with cancer, unstable angina, stroke, and hematological disorder, pediatric patients, and patients who had undergone surgery or had suffered trauma in the past 30 days were excluded from the study. Nonrelated sex and age matched as much as possible healthy individuals (*n* = 100) were also included in the study to serve as the disease-free control population. Controls were mainly hospital staffers, trainees, and unrelated healthy patient attendants.10 ml of blood sample was collected from all study participants in siliconized glass containers, containing 3.2% sodium citrate solution. Plasma was isolated within 45 min of sampling and stored at −70°C for further evaluation.

### 2.2. PAI-1 Genotyping

**Figure 1:**
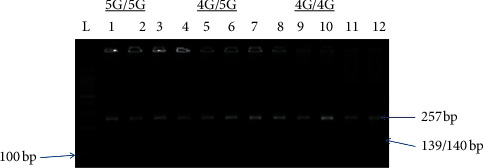
Amplified product of the 4G/5G polymorphism. L 100 bp ladder, lanes 1 to 4 for 5G allele, lanes 9 and 10 for 4G allele, and lanes 5, 6, 7, 8, 11, and 12 for 4G/5G heterozygous allele.

Genomic DNA was isolated from peripheral blood leucocytes by employing standard methods. Genotypic screening protocols for 4G/5G were done through allele-specific PCR. The PCR reaction used an upstream control primer (5′- AAG CTT TTA CCA TGG TAA CCC CTG GT- 3′), an allele-specific primer 4G (5′-GTC TGG ACA CGT GGG GA-3′) or 5G (5′-GTC TGG ACA CGT GGGGG-3′), and a common downstream primer (5′-TGC AGC CAG CCA CGT GAT TGT CTA-3′). Two PCR reactions were run per a sample (one for 4G allele and the other for 5G allele); i.e., each reaction contained upstream primer, downstream primer, and one primer for 4G or 5G. The control upstream primer is used to verify the occurrence of DNA amplification. The PCR product was electrophoresed on 1.5% agarose gel stained with ethidium bromide. A 257 bp control band resulted from the upstream and downstream primers. The 4G or 5G allele-specific primer and downstream primer produced 139 bp fragment. Patients who showed amplification products with only 4G primers were 4G/4G, and those with only 5G primers were 5G/5G and patients who had amplification products with both primers were 4G/5G. The gel for 4G/5G polymorphism is shown in Figure 1.

**Figure 2:**
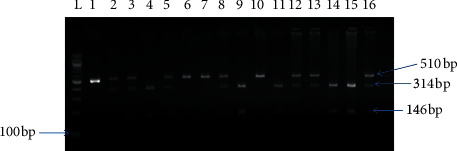
Amplified product of the -844G/A polymorphism through a PCR-RFLP. L: 100 bp ladder, lanes 1, 6, 7, and 10 homozygous mutant, lanes 2, 3, 5, 8, 12, 13, and 16: heterozygous, and lanes 4, 9, 11, 14, and 15: homozygous normal.

The -844G/A polymorphism was detected by polymerase chain reaction and restriction fragment length polymorphism (PCR-RFLP), with the use of the following primers: forward, 5′-CAGGCTCCCACTGATTCTAC-3; and reverse, 5′-GAGGGCTCTCTTGTGTCAAC-3, amplified products were digested by XhoI and separated on 2% agarose gel. The -844G allele was visualized as 314 + 146 bp bands and the -844A allele was visualized as the 510 bp fragment (Figure 2).

### 2.3. PAI-1 Antigen Assay

PAI-1 antigen was measured by a quantitative sandwich enzyme immunoassay technique (Assaypro LLC, St. Charles, USA) with a working range from 5 to 40 ng/ml. ELISA was performed strictly according to the manufacturer's instructions provided in the kit manual.

### 2.4. Statistical Analysis

Statistical analysis was performed using SPSS version 14.0 software programme. The variables between patients and controls were compared using Student's *t* test and Chi square test. Data was presented as mean ± SD or as percent of patients. Chi-square test was used for comparing genotype and allele frequencies for statistical significance between patients and controls. Odds ratios (ORs) with corresponding 95% confidence intervals (CIs) were determined to assess the strength of association of 4G/5G and -844G/A polymorphism with AMI and CSA risk. “p” values below 0.05 were considered significant.

## 3. Results

### 3.1. Clinical Characteristics of Cases (AMI and CSA) and Controls

The present study has average age 55.99 ± 10.33 years in AMI, 56.38 ± 10.55 years in CSA, and 52.07 ± 14.44 years in control group (Tables 1 and 2). A total of hundred cases were included in each group. Eighty-two percent were male in both AMI and CSA and 80% in control group. The presence of smoking was statistically significant in AMI and CSA patients as compared to controls (*p* < 0.05); however, situation was not the same in tobacco chewers (*p*=0.068). Alcohol consumption was significantly higher in individuals who suffered AMI and CSA in contrast to control subjects (*p* < 0.05). Family history of CAD has been detected in 21% and 23% of AMI and CSA cases, respectively.

Comparison among the groups with regard to HDL level was statistically significant (*p* < 0.05), reflecting in the same way between AMI and control as well as between CSA and controls (*p* < 0.05). Similarly, the differences in LDL level between AMI (109.49 ± 4.78 mg/dl) and controls (99.96 ± 3.22 mg/dl) and CSA (109.84 ± 5.19 mg/dl) and control were statistically significant (*p* < 0.05). In contrast, it is not true between AMI and CSA (*p*=1.00). BMI difference was statistically significant between CSA (23.95 ± 3.97 kg/m^2^) and controls (22.39 ± 3.92 kg/m^2^) with *p*=0.012. On the contrary, BMI difference between AMI (23.60 ± 3.53 kg/dl) and controls and with CSA was not statistically significant (*p*=0.076 and 1.0, respectively). With regard to PAI level, it is highest in case of CSA (30.04 ± 8.32 ngm/ml) followed by AMI (28.62 ± 8.57 ngm/ml) and control (22.93 ± 7.22 ngm/ml). The differences among these three groups were statistically significant (*p* < 0.05). In the same way, the comparison of AMI and CSA independently with control in terms of PAI-1 level was also statistically significant (*p* < 0.05) but not between AMI and CSA (*p* < 0.642).

### 3.2. Association of 4G/5G Polymorphism with Cases (AMI and CSA)

The distribution of genotype and allelic frequency for 4G/5G polymorphism in AMI cases and control are summarized in Table 3. With reference to 5G/5G genotype, frequency of 4G/4G and 4G/5G genotype was significantly higher in AMI cases than in control group and was found to increase the risk of AMI (OR, 6.51; 95%CI, 2.67–15.8; *p* < 0.0001 for 4G/4G; and OR, 6.52; 95% CI, 2.92–14.5; *p* < 0.0001 for 4G/5G, Table 3). The statistical analysis of observed genotypic frequencies showed significant association (*p* < 0.0001). The frequency of 4G allele was significantly higher in AMI cases than in controls (60% vs. 39%, respectively). Compared to 5G allele, the 4G allele significantly increases the risk of AMI (OR, 2.34; 95% CI, 1.57–3.50; *p* < 0.0001). We observed significant relationship between 4G/5G polymorphism and AMI risk under the dominant (OR = 6.51; 95% CI: 3.03–13.9; *p* < 0.0001) and codominant genotype (OR, 2.34; 95% CI: 1.33–4.14; *p*-0.003), whereas there is no significant relationship under the recessive model (OR, 1.79; 95% CI: 0.94–3.43; *p*-0.076).

The distributions of 4G/4G, 4G/5G, and 5G/5G genotypes were 32%, 36%, and 32% in CSA cases and 20%, 38%, and 42% in controls, respectively (Table 4). The statistical analysis of observed genotypic frequencies did not show significant difference (*p*-0.1240). With reference to 5G/5G genotype, 4G/4G genotype was significantly higher in CSA cases than in control group and increases the risk of CSA (OR, 2.10; 95%CI, 1.01–4.33; *p*-0.044, Table 4). We did not find any significant association of 4G/5G polymorphism with CSA in recessive, dominant, and codominant models.

Additionally, we also found that the genotype frequency difference between AMI and CSA cases was statistically significant (*p*-0.0001, Table 5).

### 3.3. Association of -844G/A Polymorphism with Cases (AMI and CSA)

The distributions of genotype and allelic frequency for -844G/A polymorphism in AMI and control, CSA and control, AMI and CSA are listed in Tables 6–8, respectively.

The genotype frequencies for GG, GA, and AA in AMI cases vs. control were 20%, 56%, and 24% and 24%, 54%, and 22%, respectively (Table 6). The genotype frequencies for GG, GA, and AA in the CSA cases were 18%, 54%, and 28%, while in the controls they were 24%, 54%, and 22%, respectively (Table 7). The genotype frequencies difference between AMI cases and control, CSA cases and control, AMI and CSA cases were statistically nonsignificant (*p*-0.783; *p*-0.454; *p*-0.798, respectively). Similarly, there was no significant difference in allele frequencies between AMI cases and control; CSA cases and control (*p*-0.548; *p*-0.230, respectively) (Table 8). We did not find any significant association of -844G/A polymorphism with AMI and CSA in recessive, dominant, and codominant models.

### 3.4. Association of 4G/5G and -844G/A Polymorphisms with PAI-1 Level in Cases (AMI and CSA) and Controls

The 4G/5G polymorphisms and -844G/A polymorphisms were significantly associated with increased PAI-1 levels with 4G/4G (*p*-0.019), 4G/5G (*p*-0.001) and GA (*p*-0.0009) and AA (*p*-0.022) genotypes, respectively, in AMI cases verses controls (Table 9). Likewise, elevated PAI-1 levels were significantly associated with the 4G/4G (0.003) and 4G/5G (0.0001) genotypes of 4G/5G polymorphism and GA (*p* < 0.0001) and AA (*p*-0.003) genotypes of -844G/A polymorphism in CSA cases versus control group (Table 10).

## 4. Discussion

The present study was conducted to ascertain the role of PAI-1 gene polymorphisms in the occurrence of coronary artery diseases including AMI and CSA in North-Indian population. We particularly concentrated on PAI-1 4G/5G and -844G/A polymorphisms, plasma PAI-1 levels, and their influence on development of thrombotic complications and stenosing lesions such as AMI and CSA, respectively.

Diet has significant effects on mortality due to ischemic heart disease. Various studies showed significantly reduced risk and mortality due to ischemic heart disease in individuals consuming vegetarian diet and it may be largely due to lower cholesterol concentrations in vegetarians than in nonvegetarians and due in part to other mechanisms such as reduced oxidation of LDL cholesterol and blood clotting [9, 10]. Similar to these studies, our cohort also showed significantly increased risk of AMI in nonvegetarians; on the contrary, the incidence of CSA did not increase in nonvegetarians. Hypertension and diabetes are well-known associations with the occurrence of CAD; however, we failed to obtain significant difference in the presence of CAD in hypertensive and nonhypertensive participants and more, so results showed higher incidence of CAD in nondiabetic participants [11, 12]. We also found AMI and CSA to be more often in individuals who smoke and consume alcohol and this is in congruence with previous studies [13, 14]. Advancing age and male gender are the most important known risk factors for ACS; similarly, the most common risk factor in our study was male gender followed by hypertension, nonvegetarian dietary habit, smoking, diabetes, alcohol intake, and family history of CAD [15]. Dyslipidemia includes raised LDL and TG, and low HDL poses risk to development of CAD; we, in the same way, documented significantly increased level of serum LDL and total cholesterol and low level serum HDL in patients with CSA and AMI with respect to control subjects [16–18]. The PAI-1 works to modulate the development of atherosclerosis and alter fibrinolysis. Thus, under the condition of impaired fibrinolysis, plaque rupture results in immediate formation of occlusive thrombus, leading to AMI, whereas normal or accelerated fibrinolysis prevents the formation of thrombus over ruptured atherosclerotic plaque and hence promotes the stenotic coronary lesions without precipitating thrombosis. Several studies have demonstrated the relationship of -844G/A polymorphism and PAI-1 levels with A allele and AA genotype being associated with raised level of PAI-1 protein and mRNA. We have also noted the raised PAI-1 level in individuals carrying -844GA or -844AA genotypes with higher levels in persons with higher load of A allele in all the three groups [19–21]. Few studies, as our study could not find any significant evidence to support the role of -844G/A polymorphism in AMI and CSA [22]. In contrast to that, some studies have shown association of -844G/A polymorphism and increased risk of CAD and AMI [19–23]. Although the association of AMI with the presence of 4G allele is controversial, many studies favor [23–29] and some disregard their involvement [30–32]. In our study, frequency of 4G/4G and 4G/5G genotype in AMI cases was significantly higher than controls. Thus, the presence of 4G allele does increase the risk of acute thrombosis and it has a similar reflection in terms of raised PAI-1 level with respect to the number of 4G alleles in a particular genotype. In subgroup analysis for mean plasma PAI-1 level, the PAI-1 level was significantly raised in both AMI and CSA with respect to control. CSA does not signify thrombosis; nevertheless, the level of PAI-1 was higher in CSA as compared to AMI, although not up to that significant level. This may be attributable to some of the genetic, environmental factors or other associated coronary risk factors such as insulin resistance, apart from 4G/5G or -844GA polymorphisms. We also performed subgroup analysis in diabetic and nondiabetic individuals to find out the PAI-1 level in these subgroups and the level was significantly elevated in diabetic patients as compared to nondiabetics, similar to previously conducted studies [33]. This present study is the first one to show significant association between 4G/5G allele polymorphism and risk of AMI in Indian population, suggesting the potential role of this polymorphism in development of AMI.

There were some limitations in the present study. Firstly, the sample size was small. Indian population is thought to be most diverse due to different sociocultural traditions. A single larger study with diverse sample size may help us in better understanding the association of the genetic variation of these genes with AMI and CSA risk. Further studies on larger sample size are needed to confirm our findings.

## 5. Conclusion

Along with the traditional risk factors, the 4G/5G allele polymorphism is an independent risk factor for the development of AMI. The detection of 4G/5G allele may therefore be helpful in primary prevention. Patients who carry the 4G/5G allele polymorphism have high concentrations of PAI-1, which might be involved in incidents leading to AMI. This current study for the first time revealed significant association of 4G/5G allele polymorphism with high risk of AMI in Indian population and will be helpful in identifying the genetic risk factors associated with AMI and CSA and for better management of diagnostic measures.

## Figures and Tables

**Table 1:** Clinical characteristics of patients and controls.

Characteristic	Group I (AMI) (*n* = 100)	Group II (CSA) (*n* = 100)	Group III (control) (*n* = 100)	*p* value
Age (years)	55.99 ± 10.33	56.38 ± 10.55	52.07 ± 14.44	

Gender %				0.947
Male	82	82	80	
Female	18	18	20	

Hypertension %	54	49	0	0.572
Diabetes %	24	37	0	0.065
Smoker %	36	41	11	<0.05
Alcoholic %	26	26	7	<0.05

Food habits %				0.029
Vegetarian	39	53	57	
Nonvegetarian	61	47	43	

Family history %	21	23	0	0.865
Total cholesterol(mg/dl)	168.1 ± 4.02	169.59 ± 3.80	163.9 ± 3.98	0.836
Total HDL(mg/dl)	30.18 ± 2.61	30.55 ± 2.74	41.31 ± 2.59	0.826
Total LDL(mg/dl)	109.49 ± 4.78	109.84 ± 5.19	99.96 ± 3.22	<0.005
BMI	23.60 ± 3.53	23.97 ± 3.97	22.39 ± 3.91	0.452
PAI-1 antigen (ng/ml)	28.62 ± 8.57	30.04 ± 8.32	22.93 ± 7.22	0.199

Data presented as mean ± SD except gender, hypertension, diabetes, smoker, alcoholic, food habits, and family history. ^*∗*^Significant at *p* < 0.05. BMI: body mass index; HDL: high density lipoprotein; LDL: low density lipoprotein; PAI: plasminogen activator inhibitor-1.

**Table 2:** PAI level (ng/nl) in diabetic and nondiabetic cases.

Group	Diabetic			Nondiabetic			*p* value
	*N*	Mean (SD)	PAI level (ng/ml)	*N*	Mean (SD)	PAI level (ng/ml)	
Group I (AMI) (100)	24	33.75 (7.93)	32.5	76	27 (8.16)	27.5	<0.05
Group II (CSA) (100)	37	35.62 (6.67)	37	63	26.7 (7.43)	27	<0.05
Group III (control) (100)	0	0	0	100	22.9 (7.21)	25	<0.05

PAI: plasminogen activator inhibitor-1.

**Table 3:** Distribution of genotype and allelic frequency and association analysis of 4G/5G polymorphism with AMI cases under different genetic models.

Genotype/allele		Group I (AMI) (*n* = 100)	Group III (control) (*n* = 100)	Odds ratio (95% CI)	*p* value
4G/4G		31 (31%)	20 (20%)	6.51 (2.67–15.8)	<0.0001^*∗*^
4G/5G		59 (59%)	38 (38%)	6.52 (2.92–14.5)	<0.0001^*∗*^
5G/5G		10 (10%)	42 (42%)	Ref	Ref
		*p* value < 0.0001			
Recessive model	4G/4G	31	20	1.79 (0.94–3.43)	0.076
	4G/5G + 5G/5G	69	80		
Dominant model	4G/5G + 4G/4G	90	58	6.51 (3.03–13.9)	<0.0001^*∗*^
	5G/5G	10	42		
Codominant model	4G/5G	59	38	2.34 (1.33–4.14)	0.003^*∗*^
	5G/5G + 4G/4G	41	62		

*Allele*					
4G		120 (60%)	78 (39%)	2.34 (1.57–3.50)	<0.0001^*∗*^
5G		80 (40%)	122 (61%)		

OR: odds ratio, CI: confidence interval, and *n*: number in sample. ^*∗*^Significant at *p* < 0.005.

**Table 4:** Distribution of genotype and allelic frequency and association analysis of 4G/5G polymorphism with CSA under different genetic models.

Genotype/allele		Group II (CSA) (*n* = 100)	Group III (control) (*n* = 100)	Odds ratio (95% CI)	*p* value
4G/4G		32 (32%)	20 (20%)	2.10 (1.01–4.33)	0.044^*∗*^
4G/5G		36 (36%)	38 (38%)	1.24 (0.65–2.37)	0.509
5G/5G		32 (32%)	42 (42%)	Ref	Ref
		*P* value—0.1240			
Recessive model	4G/4G	32	20	1.88 (0.98–3.58)	0.054
	4G/5G + 5G/5G	68	80		
Dominant model	4G/5G + 4G/4G	68	58	1.53 (0.86–2.74)	0.144
	5G/5G	32	42		
Codominant model	4G/5G	36	38	0.91 (0.51–1.62)	0.769
	5G/5G + 4G/4G	64	62		

*Allele*					
4G		100 (50%)	78 (39%)	1.56 (1.05–2.32)	0.027^*∗*^
5G		100 (50%)	122 (61%)		

OR: odds ratio, CI: confidence interval, and *n*: number in sample. ^*∗*^Significant at *p* < 0.05.

**Table 5:** Genotypic distribution of 4G/5G polymorphism for AMI and CSA cases.

Genotype	Group I (AMI) (*n* = 100)	Group II (CSA) (*n* = 100)	*p* value
4G/4G	31 (31%)	32 (32%)	0.0001^*∗*^
4G/5G	59 (59%)	36 (36%)	
5G/5G	10 (10%)	32 (32%)	

^*∗*^Significant at *p* < 0.05.

**Table 6:** Distribution of genotype and allelic frequency and association analysis of -844G/A polymorphism with AMI under different genetic models.

Genotype/allele		Group I (AMI) (*n* = 100)	Group III (Control) (*n* = 100)	Odds ratio (95% CI)	*p* value
GG		20 (20%)	24 (24%)	Ref	Ref
GA		56 (56%)	54 (54%)	1.24 (0.61–2.50)	0.541
AA		24 (24%)	22 (22%)	1.30 (0.57–2.99)	0.524
		*p* value—0.783			
Recessive model	AA	24	22	1.11 (0.57–2.16)	0.736
	GA + GG	76	78		
Dominant model	GA + AA	80	76	1.26 (0.64–2.47)	0.495
	GG	20	24		
Codominant model	GA	56	54	1.08 (0.62–1.89)	0.776
	GG + AA	44	46		

*Allele*					
G		96 (48%)	102 (51%)	1.12 (0.76–1.66)	0.548
A		104 (52%)	98 (49%)		

OR: odds ratio, CI: confidence interval, and *n*: number in sample. ^*∗*^Significant at *p* < 0.05.

**Table 7:** Distribution of genotype and allelic frequency and association analysis of -844G/A polymorphism with CSA under different genetic models.

Genotype/allele		Group II (CSA) (*n* = 100)	Group III (control) (*n* = 100)	Odds ratio (95% CI)	*p* value
GG		18 (18%)	24 (24%)	Ref	Ref
GA		54 (54%)	54 (54%)	1.33 (0.65–2.73)	0.432
AA		28 (28%)	22 (22%)	1.69 (0.74–3.88)	0.210
		*p* value 0.454			
Recessive model	AA	28	22	1.37 (0.72–2.62)	0.328
	GA + GG	72	78		
Dominant model	GA + AA	82	76	1.43 (0.72–2.85)	0.298
	GG	18	24		
Codominant model	GA	54	54	1.00 (0.57–1.74)	1.000
	GG + AA	46	46		

*Allele*					
G		90 (45%)	102 (51%)	1.27 (0.85–1.88)	0.230
A		110 (55%)	98 (49%)		

OR: odds ratio, CI: confidence interval, and *n*: number in sample. ^*∗*^Significant at *p* < 0.05.

**Table 8:** Genotype distribution of -844G/A polymorphism for AMI and CSA.

Genotype	Group I (AMI) (*n* = 100)	Group II (CSA) (*n* = 100)	*p* value
GG	20 (20%)	18 (18%)	0.798
GA	56 (56%)	54 (54%)	
AA	24 (24%)	28 (28%)	

^*∗*^Significant at *p* < 0.05.

**Table 9:** Comparison of PAI level (ng/ml) according to 4G/5G and -844G/A polymorphism in the AMI cases and control groups.

Genotype	AMI		Control		*p* value
	Number	Mean (SD)	Number	Mean (SD)	
4G/4G	31	28.62 (8.57)	20	23.04 (7.23)	0.019^*∗*^
4G/5G	59	28.40 (8.38)	38	22.94 (7.20)	0.001^*∗*^
5G/5G	10	25.63 (11.41)	42	22.93 (7.21)	0.349
GG	20	25.43 (11.15)	24	22.71 (7.33)	0.337
GA	56	28.12 (8.21)	54	23.11 (7.08)	0.0009^*∗*^
AA	24	28.62 (8.57)	22	23.04 (7.23)	0.022^*∗*^

^*∗*^Significant at *p* < 0.05.

**Table 10:** Comparison of PAI level (ng/ml) according to 4G/5G and -844G/A polymorphism in the CSA cases and control groups.

Genotype	CSA		Control		*p* value
	Number	Mean (SD)	Number	Mean (SD)	
4G/4G	32	30.04 (8.32)	20	23.04 (7.23)	0.003^*∗*^
4G/5G	36	30.33 (7.57)	38	22.94 (7.20)	0.0001^*∗*^
5G/5G	32	29.56 (8.08)	42	22.93 (7.21)	0.0004^*∗*^
GG	18	26.48 (9.51)	24	22.71 (7.33)	0.154
GA	54	30.21 (7.54)	54	23.11(7.08)	<0.0001^*∗*^
AA	28	30.04 (8.32)	22	23.04 (7.23)	0.003^*∗*^

^*∗*^Significant at *p* < 0.05.

## Data Availability

Data may be restricted in order to protect patient privacy. It can be obtained from the corresponding author on request only after institution approval.

## Abbreviations

AMI: Acute myocardial infarction
CSA: Chronic stable angina
PAI-1: Plasminogen activator inhibitor-1
CAD: Coronary artery disease
USA: Unstable angina
PCR-RFLP: Polymerase chain reaction-restriction fragment length polymorphism
ACC: American College Cardiology
AHA: American Heart Association
OR: Odds ratio
CI: Confidence interval.

## Additional Points

(i) There is positive association of 4G/5G and -844G/A polymorphisms with increased level of PAI-1 in patients with CSA and AMI. (ii) -844G/A polymorphism failed to show any significant association with the risk of development of AMI and CSA. (iii) There is significant association of 4G/5G polymorphism with increased AMI risk. (iv) This study will be helpful in identifying the genetic risk factors associated with AMI and CSA in a population.
